# Mitochondrial genomes illuminate the evolutionary history of the Western honey bee (*Apis mellifera*)

**DOI:** 10.1038/s41598-020-71393-0

**Published:** 2020-09-03

**Authors:** Erik Tihelka, Chenyang Cai, Davide Pisani, Philip C. J. Donoghue

**Affiliations:** 1 Department of Animal Science, Hartpury College, Hartpury, GL19 3BE UK; 2 State Key Laboratory of Palaeobiology and Stratigraphy, Nanjing Institute of Geology and Palaeontology, and Centre for Excellence in Life and Paleoenvironment, Chinese Academy of Sciences, Nanjing, 210008 China; 3 School of Earth Sciences, University of Bristol, Life Sciences Building, Tyndall Avenue, Bristol, BS8 1TQ UK; 4 School of Biological Sciences, University of Bristol, Life Sciences Building, Tyndall Avenue, Bristol, BS8 1TQ UK

**Keywords:** Phylogenetics, Entomology

## Abstract

Western honey bees (*Apis mellifera*) are one of the most important pollinators of agricultural crops and wild plants. Despite the growth in the availability of sequence data for honey bees, the phylogeny of the species remains a subject of controversy. Most notably, the geographic origin of honey bees is uncertain, as are the relationships among its constituent lineages and subspecies. We aim to infer the evolutionary and biogeographical history of the honey bee from mitochondrial genomes. Here we analyse the full mitochondrial genomes of 18 *A. mellifera* subspecies, belonging to all major lineages, using a range of gene sampling strategies and inference models to identify factors that may have contributed to the recovery of incongruent results in previous studies. Our analyses support a northern African or Middle Eastern origin of *A. mellifera*. We show that the previously suggested European and Afrotropical cradles of honey bees are the result of phylogenetic error. Monophyly of the M, C, and O lineages is strongly supported, but the A lineage appears paraphyletic. *A. mellifera* colonised Europe through at least two pathways, across the Strait of Gibraltar and via Asia Minor.

## Introduction

As probably the single most significant pollinator of agricultural crops and wild plants and as a producer of a variety of foodstuffs with nutritional, medical, and cosmetic uses such as honey and propolis, the importance of understanding the evolutionary history of *Apis mellifera* Linnaeus, 1758 cannot be overstated. While the Western honey bee is native to Europe, Africa, the Middle East, and parts of Asia^1^, the ability of *A. mellifera* to colonise virtually all habitable biomes on Earth and adapt to diverse bioclimatic conditions is a living proof of the specie’s remarkable morphological and behavioural plasticity^2^. To date, over 30 separate subspecies (or ‘geographical races’) have been described^3–7^. The distinctiveness of subspecies is in many cases not readily apparent by examining live or pinned individuals and identification has to be carried out based on quantitative morphometric^6,8,9^ or molecular analyses^5,10,11^. The classification of honey bee subspecies has important practical implications for apiculture. Beekeepers have long recognised that bee races differ in a number of behavioural traits such as calmness, swarming intensity, honey production, ability to utilize different sources of forage, and resistance to disease^6,12–15^. A pan-continental study of European subspecies has shown that locally-adapted stock enjoys better survival rates and may have lower pathogen levels^16,17^, highlighting the importance of conserving the genetic diversity of native honey bee populations^7,18^.

A reliable backbone phylogeny of the Western honey bee would allow apidologists to study the evolution of economically important traits in this species and the basis of its adaptation to different environmental conditions^19^. Although more gene sequences from across the entire native range of *A. mellifera* are available now than ever before^20^, a number of long-standing questions has still not been answered. Notably, the geographic origin of the Western honey bee has historically been a subject of controversies^1,21,22^. The most recent whole genome analysis has lent support to either a north eastern African or a Middle Eastern origin of *A. mellifera*^23^, but discriminating between these two hypotheses has been proven challenging using any dataset. In stark contrast, analyses of complete mitochondrial genomes have recovered *A. m. mellifera* as the basalmost subspecies, thus placing the origin of honey bees within northern Europe^24–30^. A European origin of honey bees does not seem unreasonable, as the oldest unequivocal fossil representatives of the genus *Apis* are known from the Oligocene of France and Germany, and fossil European honey bees also show high degrees of morphological disparity^4,31–34^. On the other hand, given that all other living species of the genus *Apis* occur in Asia, the idea that *A. mellifera* may have originally dispersed from the east has enjoyed lasting popularity since the 1950s^1,35–38^. Unexpectedly, support for an origin of honey bees in tropical Africa was recovered by an analysis of 1,136 SNPs from 341 bees^2^. Notably, most recent phylogenomic studies investigating honey bee origins^1,2,39^ have relied on the neighbour joining (NJ) algorithm which is prone to systematic errors and can yield misleading topologies^40^.

The relationships among the individual subspecies of *A. mellifera* are likewise unclear, with studies yielding ambigious results. Traditionally, the Western honey bee has been divided into four evolutionary lineages: the A lineage (subspecies native to Africa), the M lineage (western and northern Europe), the C lineage (southern and eastern Europe), and the O lineage (Caucasus, Turkey, Middle East, Cyprus, Crete) based on morphometric and molecular data^2,6,41–43^. In recent years, two new purportedly isolated lineages have been proposed based on molecular data. Honey bees from Ethiopia deviate substantially from the A lineage into which they have been originally placed and were thus referred to a distinct group of their own, the Y lineage^44^. A sixth lineage from Syria and Lebanon has been identified as clearly divergent from the O lineage based on neighbour joining of microsatellite loci and mitochondrial DNA^45,46^. Here we refer to this proposed group as the S lineage. Moreover, some studies do not recognise the distinction between the C and O lineages^21,38,47^.

To shed light on the controversial inter-relationships among honey bee subspecies and to provide a reliable backbone phylogeny of *A. mellifera*, we used a collection of recently sequenced complete mitochondrial genomes representing more than half of the described honey bee subspecies from across its entire native range. We analysed our data with methods that allow for the identification of sources of phylogenetic incongruence, utilizing different gene sampling strategies and inference models.

## Material and methods

### Sequence data

The mitochondrial genome of *A. mellifera* consists of around 16,463 bp and includes 13 protein coding genes, 22 transfer RNA (tRNA) genes, two ribosomal (rRNA) genes, and one control region^48^. All mitochondrial genomes of *A. mellifera* sequenced to date were obtained from GenBank in January 2020 alongside with the mitochondrial genomes of *A. cerana, A. florea,* and *A. dorsata*, which were used as outgroups. In total, 18 *A. mellifera* mitogenomes were analysed, representing more than half of its known subspecific diversity. GenBank accession numbers are provided in Table S1.

Data for the 13 protein coding genes and two RNA molecules were downloaded, aligned, and concatenated using PhyloSuite v1.2.1^49^. Protein-encoding genes were unambiguously aligned owing to few gaps and their codon-based structure using the g-ins-i algorithm implemented in the mafft v 7.313 plugin^50^. The ribosomal RNAs 16S and 18S were aligned in mafft using the e-ins-i algorithm.

### Phylogenomic analyses

To test the effects of gene sampling on topology recovery, we prepared three datasets: the first and second codon positions only (P12), P12 and the two rRNA genes (P12RNA), and all codon positions together (P123). The third codon position of the protein-coding genes has been shown to suffer high degrees of saturation, which can potentially lead to biased phylogenies, and as such its exclusion is recommended to reduce data heterogeneity^51,52^.

To examine potential sources of phylogenomic conflict resulting from inappropriate model selection, we analysed the sequences using both site-heterogeneous Bayesian inference (BI) and site-homogeneous maximum likelihood (ML) methods. The inappropriate selection of inference models represents one of the key sources of phylogenomic error^53^. In particular, high compositional and rate heterogeneity of sequences can result into simpler site-homogeneous models yielding results affected by systematic error such as long branch attraction^54,55^. Such types of error are often difficult to notice, because they may be strongly supported and recovered consistently^56^. On the other hand, more complex site-heterogeneous models that account for compositional and rate heterogeneity generally fit data better and have consequently been applied to resolving difficult phylogenomic problems such as the origin of eukaryotes or the basal branching order in Metazoa^54,55,57–63^.

The BI site-heterogeneous mixture model CAT-GTR + G was run in PhyloBayes mpi 1.7^64^. Two independent Markov chain Monte Carlo (MCMC) chains were run until convergence (maxdiff < 0.3). For each PhyloBayes run, we used the bpcomp program to generate output of the largest (maxdiff) and mean (meandiff) discrepancy observed across all bipartitions.

For the ML analyses, the concatenated datasets partitioned by codon were analysed with partitionfinder 2.1.1 to select the most appropriate models^65^. The ‘greedy’ algorithm was used to search ‘all’ models under the Bayesian information criterion, with branch lengths unlinked. The partitioning scheme is reported in Table S2. ML analyses using the selected models were performed in iq-tree v 1.6.12. Analyses were run using 1,000 ultra-fast bootstraps^66^.

All illustrations were prepared with iTOL v. 5.5.1^67^, Adobe Photoshop v. 21.2, and Adobe Illustrator v. 24.2.

## Results

**Figure 1:**
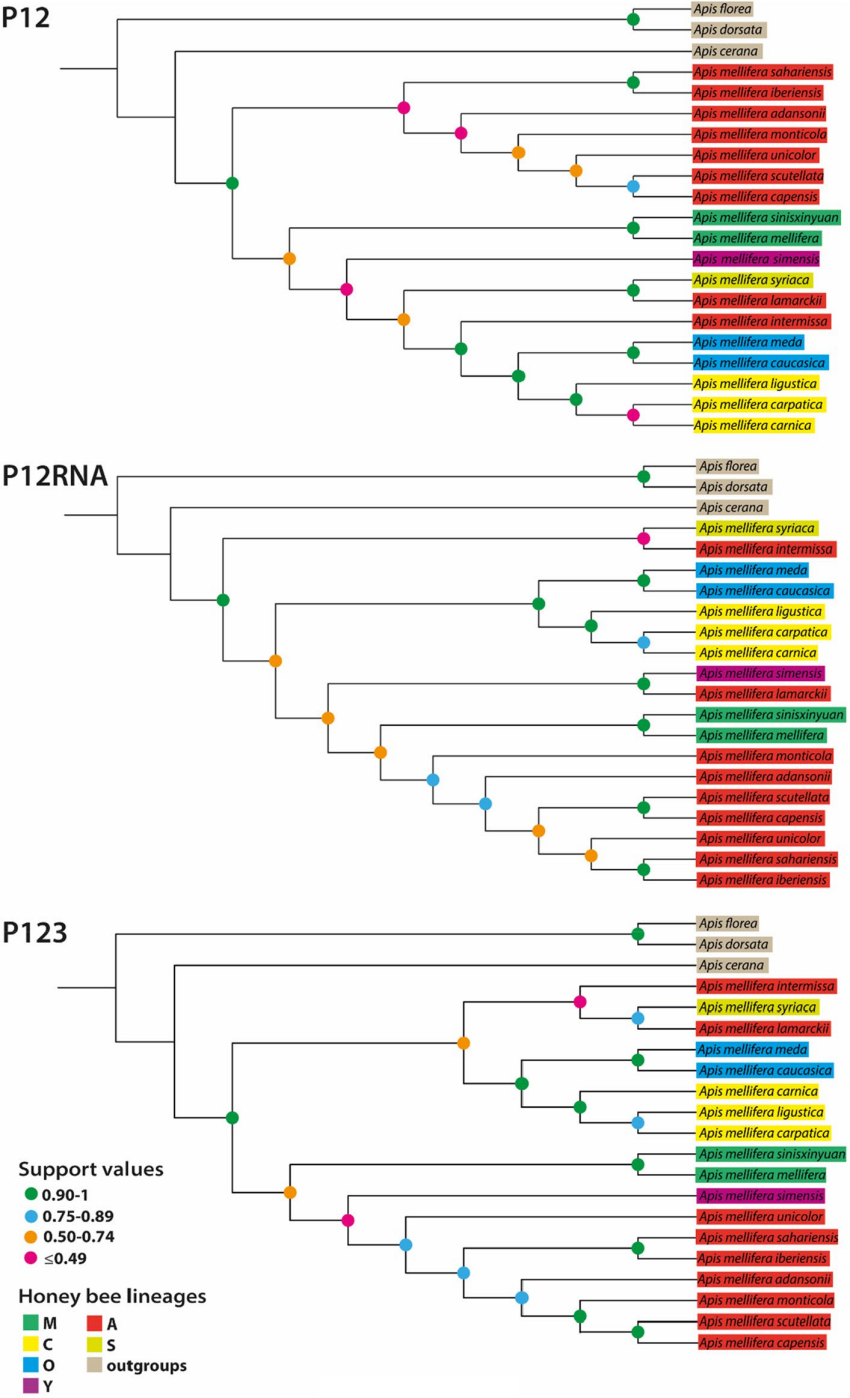
Three competing topologies recovered based on different gene sampling approaches with the site-heterogeneous CAT-GTR + G model ignoring branch length for easier legibility. Subspecies are grouped into proposed molecular lineages primarily after Ruttner^6^ and Meixner et al.^5^. Support values represent Bayesian posterior probabilities. Abbreviations: P12, first and second codon positions of protein coding mitochondrial genes; P12RNA, first and second codon positions of protein coding mitochondrial genes and two ribosomal (rRNA) genes; P123, protein coding mitochondrial genes without the third codon position excluded.

The largest discrepancies (maxdiff) in all PhyloBayes runs were < 0.3, indicating they all represent phylogenetically informative runs^68^. Trees recovered with the CAT-GTR + G model are displayed in Fig. 1, trees with computed branch lengths and topologies recovered with the site-homogeneous ML models are presented in Figs. S1–S12. A phylogenetic hypothesis based on the CAT-GTR + G analysis of P12RNA is presented as the graphical abstract. Shallow relationships among subspecies typically received high support, although the same was not always the case for deeper nodes, as has been typical of most honey bee phylogenies conducted to date^7,20^.

Our analyses using the site-heterogeneous CAT-GTR + G model always recovered subspecies from northern Africa and the Middle East as sister to the remaining *A. mellifera* subspecies. Full support (Bayesian Posterior Probabilities [BPP] = 1) was recovered for the P12RNA dataset where *A. m. syriaca* and *A. m. intermissa* formed the basalmost clade. The former is native to the eastern Mediterranean region while the latter occurs in Tunisia, Algeria, and in Morocco between the Atlas Mountains and the Mediterranean and Atlantic coasts^69,70^. Analyses of the P12 and P123 datasets have recovered *A. m. sahariensis* (northwest Africa.) + *A. m. iberiensis* (Iberian Peninsula), and *A. m. syriaca* (Near East and Israel) + *A. m. intermissa* (northern Africa) as the basalmost subspecies, respectively, albeit with low support.

ML analyses of P12 and P12RNA have recovered the northern African *A. m. intermissa* as the basalmost subspecies with full support (Maximum Likelihood Bootstrap [MLB] = 100, Figs. S2, S4). A clade comprising *A. m. mellifera* and *A. m. sinisxinyuan* was recovered as sister to the remaining honey bees in the ML analysis of P123 (MLB = 100, Fig. S6).

Regardless of the dataset analysed or the model used, the monophyly of the M lineage was always recovered with maximal support (BPP = 1, MLB = 100). Likewise, lineages C and O were always monophyletic and together formed a monophyletic clade. Notably, the A lineage was never monophyletic in the PhyloBayes analyses, with a separation between the basal northern African subspecies and the more derived Afrotropical ones.

## Discussion

### The cradle of Western honey bees: Asia, Africa, Middle East, or Europe?

Disentangling the phylogeny of *A. mellifera* has long been hampered by the limited availability of molecular data for the majority of honey bee subspecies. Molecular analyses have traditionally recovered conflicting relationships among honey bee races, resulting into uncertainties over the geographic origin of the species^1^.

An Asian origin of honey bees seems rather intuitive and it is probably for this reason that it has long enjoyed support in apidological circles^1,35–37^. Of the ten or so recognised species of the genus *Apis*, all except for *A. mellifera* occur in Asia^71^, so it would be reasonable to expect that this region is also the historical centre of the Western honey bee. The native range of *A. mellifera* in Asia includes Kazakhstan, Mongolia, the south of Russia, and a distinct *A. mellifera* subspecies further occurs in China^3,72^. Even though most recent genomic analyses of honey bees have failed to recover an Asian cradle of the Western honey bee, it has still been regarded as the most parsimonious explanation^1,39^; after all, the basal Asian populations may have simply gone extinct or were not sequenced yet. However, we caution that the evolutionary history of the genus *Apis* may be considerably more complex. Although the oldest members of the genus *Apis* are known from the Oligocene of Western Europe, these species are not necessarily basal and are morphologically similar to the extant giant honey bee *A. dorsata* native to south and southeast Asia^32–34^. This indicates that the genus *Apis* may have been present in Europe long before the origin of *A. mellifera*, and so there is no reason to expect an Asian origin of the species*.*

The hypothesis of honey bee genesis in tropical or subtropical Africa is relatively recent and has been proposed by the first SNP analysis of honey be relationships by Whitfield et al.^2^. However, extensive reanalyses of Whitfield and colleagues’ original dataset excluding samples with potentially hybrid origins^1^ as well as analyses of new SNPs^39^ have failed to find unequivocal support for the ‘out of Africa’ hypothesis. It is notable that all three studies^1,2,39^ used the neighbour joining (NJ) algorithm to infer evolutionary relationships. While NJ is computationally superfast and can be used to give a quick reference tree, it is also prone to systematic errors^40^ and as such is not generally recommended for phylogenomic studies^56^. Analyses that have used ML inference methods have rejected a deep African origin of *A. mellifera*^23^. Our analyses with the CAT-GTR + G model, that has been designed to specifically counter the effects of systematic error, also never recovered an Afrotropical origin of Western honey bees. It therefore seems that the ‘out of Africa’ result is a phylogenetic artefact probably caused by a systematic error.

Our analyses have recovered northern African subspecies, and occasionally the Middle Eastern *A. m. syriaca*, as the basalmost Western honey bees. This result is congruent with the largest molecular phylogeny based on whole honey bee genomes from across the species’ native range^23^ and with Ruttner’s et al*.*^21^ classical morphometric analysis of 33 characters on 404 bees. However, this finding is in contrast with ML analyses of honey bee mitochondrial genomes which have consistently recovered the dark European honey bee (*A. m. mellifera*, M lineage) as the basalmost subspecies. Analyses that have recovered a European cradle of *A. mellifera* have invariably used site-homogeneous models^24–30^. In our analyses, we have only recovered the M lineage as the basalmost group when the P123 dataset was analysed under ML. The same result was not recovered with ML analyses of the P12 and P12RNA datasets excluding the heterogeneous third codon position, nor when the data were analysed with the CAT-GTR + G model. Since the European origin of honey bees is only recovered in analyses that do not account for the biasing effects of data compositional heterogeneity, we conclude that it is most likely artefactual.

Overall, the origins of *A. mellifera* appear to lie in northern Africa or the Middle East. Distinguishing between these two regions is difficult at the present stage since only a few mitochondrial genomes of local bee races have been sequenced. The sister relationship between the African *A. m. intermissa* and the Middle Eastern *A. m. syriaca*, which have been recovered as the basalmost clade in our CAT-GTR + G analysis of P12RNA, suggests that the ancestral range of *A. mellifera* possibly encompassed both regions. Therefore, to narrow down the geographical origin of the Western honey bee, data for more subspecies occurring in the region will be required.

### Relationships among *Apis mellifera* subspecies

Given the meagre amount of morphological variation among honey bee subspecies, they have historically been defined primarily by quantitative measurements of around 40 principal characters such colour, pilosity, wing venation characters, and the sizes of various body parts^6^. Unfortunately, quantitative morphological characters are difficult to analyse phylogenetically^73^ and suffer from widespread homoplasy ^74^. Nevertheless, our analysis recovered strong support for three out of the four morphologically defined honey bee lineages: O, C, and M. The lineages O and C formed a strongly supported monophyletic group, an affinity already suggested by morphometry^6^ and earlier analyses of mitochondrial markers^38,74^. The two M lineage subspecies, the European *A. m. mellifera* and the Chinese *A. m. sinisxinyuan*, were also recovered in all analyses. The apparent paraphyly of the A lineage is difficult to interpret, as it was not recovered with high support. Nonetheless, the separation of northern and south African honey bees into two distinct clades has previously been detected in analyses of a limited number of mitochondrial markers^44,75^, and so the problem requires further investigation.

Aside from the four traditional morphological groups, molecular studies have suggested the existence of at least two additional lineages. The Y lineage has been recognised as a distinctive grouping of honey bees from Ethiopia based on parsimony analyses of microsatellite markers and mitochondrial DNA^44^. The distinctiveness of Ethiopian bees, traditionally placed within the A lineage, has further been demonstrated by pheromone^76^ and morphometric analyses^77,78^, although the latter also found a high degree of introgression between Ethiopian populations and the neighbouring well-defined African subspecies. We found a close proximity between the Ethiopian *A. m. simensis* and part of the paraphyletic A lineage, clearly separating it from the geographically close O lineage. These results are in line with the ML analysis of Cridland et al.^23^. The phylogenetic position of *A. m. simensis* was poorly supported or not supported all in our CAT-GTR + G analyses. Therefore, the validity of *A. m. simensis* as a separate lineage cannot be rejected at present but is contingent upon future validation.

The S lineage, consisting of *A. m. syriaca*, has been considered as a separate bee lineage by Alburaki et al.^46^. This subspecies occurs throughout Syria, Lebanon and north Jordan and has been assigned to the A lineage based on morphological characters^6,21^, although a mitochondrial analysis placed it within the O lineage^42^. The position of the Syrian honey bee has been recovered with no or little support, although a sister relationship with *A. m. lamarckii* was found in the CAT-GTR + G analysis of the P12 dataset (BPP = 0.93, Fig. 1). As such the phylogenetic position of the Syrian bee remains an open question.

### Biogeographic history of Western honey bees

**Figure 2:**
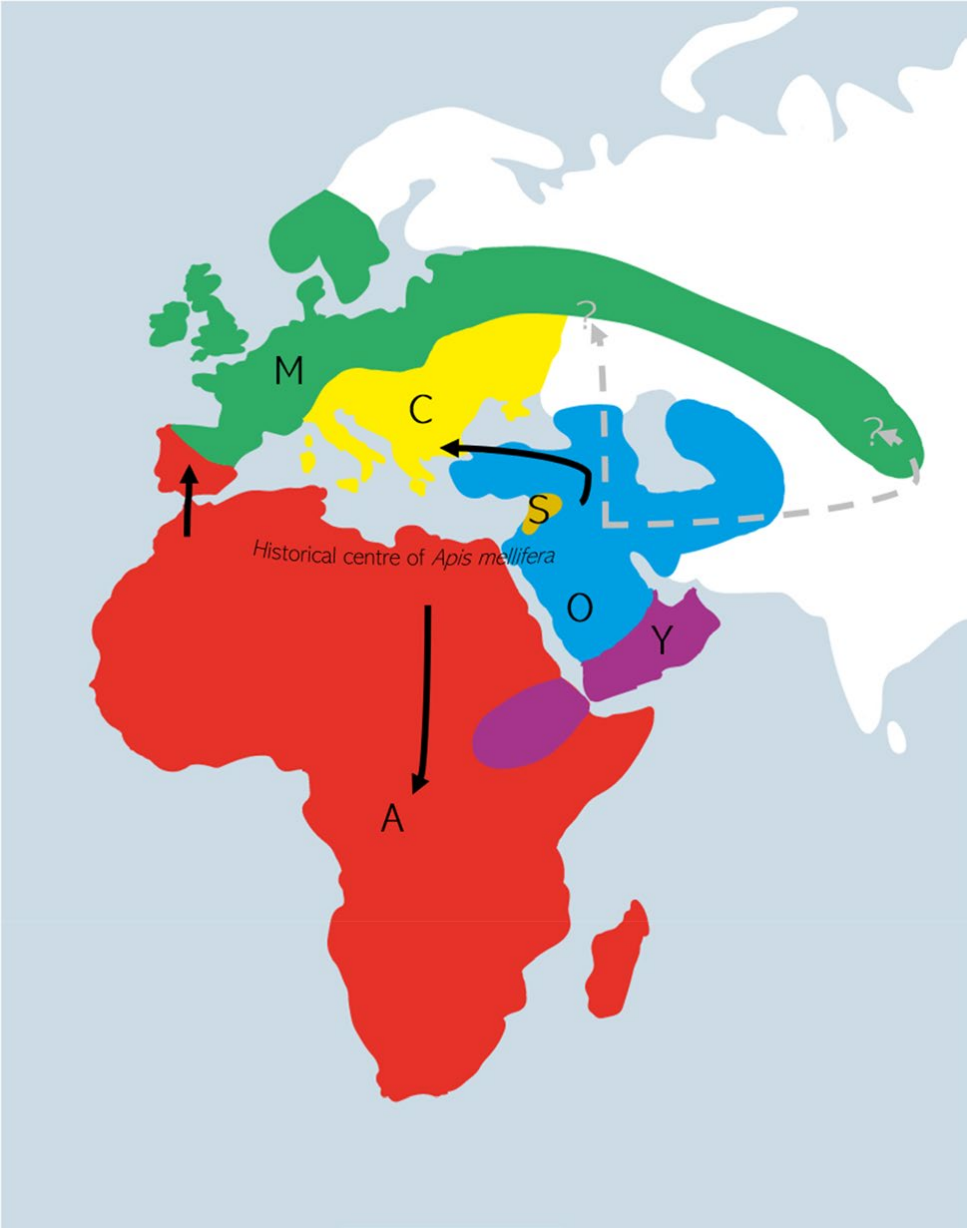
Hypothesis of *Apis mellifera* origin and dispersal routes. Solid black arrows are based on well-supported relationships inferred from Bayesian analysis of mitochondrial genomes while dashed grey arrows represent hypothetical dispersal routes. Lineage ranges are based largely on Ruttner^6^ and Dogantzis and Zayed^20^.

Regardless of the dataset analysed, our Bayesian analyses consistently recovered a strongly supported sister group relationship between the closely co-occurring *A. m. sahariensis* native to the oasis regions of the Sahara Desert and *A. m. iberiensis* from Spain and Portugal (Fig. 1). The Iberian honey bee was nested within the A lineage in our analyses regardless of the dataset analysed. While it has been placed into the M lineage based on morphological characters^6,79,80^, molecular analyses indicate that the group has a significant degree of admixture with the A lineage, especially in the south of the Iberian peninsula, and consequently it has been assigned to the A lineage by some authors^5,38,81,82^. This indicates that a part of the ancestral northern African honey bee population probably colonised Europe across the Strait of Gibraltar. This hypothesis has been proposed by morphometric analyses^21^ but was questioned by early analyses based on mitochondrial markers^38,74^. It should be noted that these analyses used parsimony and distance methods, which are outperformed by model-based BI and ML methods^83,84^. We therefore consider a cross-Gibraltar dispersal from northern Africa to Europe as highly likely (Fig. 2).

A second migration route from northern Africa and the Middle East to Europe likely occurred via Asia Minor or the Caucasus. This is suggested by the strongly supported clade comprising the lineages O and C (Fig. 1). While the basal O lineage bees occur in the Near East and Caucasus, the more derived C lineage bees are native to southern Europe. Whether honey bees dispersed into Europe via Turkey or also crossed the Caucasus and North Asia remains to be determined, as whole mitochondrial sequences are not available yet for the phylogenetically important subspecies *A. m. anatolica, A. m. macedonica,* and *A. m. cecropia.* We tentatively consider the former as more likely, since a cross-Turkish dispersal is supported by morphometric data^21^. Moreover, a distance analysis of *ND2* sequences recovered the Turkish *A. m. anatolica* as sister to the Near Eastern *A. m. meda*^85^.

The dispersal of M lineage honey bees into Europe and Asia is hard to explain, since the position of the M lineage was recovered as close to A lineage bees in CAT-GTR + G analyses, but without strong support. Chen et al.^3^ suggested that *A. m. mellifera* and *A. m. sinisxinyuan* are close to O and C lineage bees based on a NJ analysis. This result would imply that the origin of the M lineage lies in Asia Minor or the Near East. Clearly, the origin and position of the M linage warrants further study.

Understanding what historical drivers controlled the dispersal of Western honey bees is difficult. The only unequivocal evidence about the timing of evolutionary events can be obtained from the fossil record. Ideally, it would be possible to use subfossil remains of honey bees identified to subspecies level to calibrate the *A. mellifera* phylogeny^86^, which would allow us to correlate splitting events within the tree with known bioclimatic events. Unfortunately, the subfossil record of *A. mellifera* is scant. Inclusions in copal are often cited as the oldest subfossils of Western honey bees, but the copal itself is of uncertain provenance^87–89^. Copal is moreover notoriously difficult to date and may be anywhere from 5 million to several years old^90^. Other honey bee remains are only known from archaeological sites, at most several thousand years old^91,92^. As such, studies have so far yielded highly divergent results; the split between *A. cerana* and *A. mellifera* has been variously estimated as having occurred between 6 and 25 million years ago^38,72,93,94^ and the rapid divergence between *A. mellifera* subspecies between 0.3 and 1.3 million years ago^6,38,75^. Wallberg et al.^39^ used the genealogical concordance method to estimate that honey bee subspecies diverged between 13,000–38,000 years ago, which would correspond to the last glacial maximum, implying that the expansion of *A. mellifera* from its region of origin into Europe began after the retreat of the ice sheets. The expansion of the honey bee into the Afrotropics may have been controlled by climate-induced desertification and vegetation shifts in the Pleistocene^75^. However, it is clear that the timescale of honey bee diversification will have to be revisited with more subfossil at hand to verify these hypotheses.

## Future directions

While mitochondrial genomes provide a useful insight into the evolutionary history of the Western honey bee, several caveats must be pointed out. Only slightly more than half of the currently recognised honey bee subspecies have their mitochondrial genomes sequenced and some genomes from the phylogeographically important Middle East and Asia Minor are still lacking. This means that our understanding of honey bee origin and dispersal is still correspondingly incomplete.

The low support values recovered at some nodes could possibly result from the fact that the genomes of closely related honey bee subspecies are usually very similar^1,23^. Bootstrap support for groups is calculated as the proportion of times a given clade is recovered when a subset of the data is resampled^95^. As such, bootstrap support does not necessarily reflect phylogenetic signal, but assesses data redundancy, which is expected to be low in sequences with low variability^96^. Similarly, Bayesian posterior probabilities appear low when analysing sequences of potentially hybrid origin^97^. We expect that better supported topologies can be obtained by increasing gene sampling in future analyses.

Natural and human-induced hybridisation of honey bee populations has resulted into a considerable degree of genetic admixture among the subspecies^98–102^. This is the case for feral and managed bee populations within the Old World as well as for populations imported into the Americas and Australia^103,104^. As a result, most honey bee subspecies have not experienced long periods of isolation^1^ and the evolutionary trees produced by us represent only pragmatic approximations of honey bee evolutionary history. Ultimately, it may be more appropriate to think of honey bee evolution as a web of intertwining populations rather than a strictly dichotomous branching tree. However, disentangling the honey bee ‘evolutionary web’ is likewise contingent upon obtaining more whole genome sequences from across its native range.

## Conclusions

1. The Western honey bee originated in northern Africa or the Near East. This conclusion is congruent with morphological^21^ and the most extensive molecular^23^ datasets.
2. Earlier hypotheses on an Afrotropic^2^ or European^27^ origin of *A. mellifera* appear to be erroneous. They are never recovered when methods for countering the effects of rate heterogeneity are applied to datasets.
3. *A. mellifera* colonised Europe from northern Africa or the Near East via at least two routes: across the Strait of Gibraltar and via Turkey, although a possible route via the Caucasus or North Asia is subject to validation once more genomes of subspecies native to these areas are sequenced.
4. The A lineage has been recovered as paraphyletic, split between north and south African subspecies, albeit with low support.
5. The O and C lineages form a strongly supported monophyletic group in all analyses. The basal O lineage bees are native to the Near East and Caucasus, while the more recently diverged C lineage bees occur in southern Europe.
6. *A. m. mellifera* is grouped together with the Chinese *A. m. sinisxinyuan*. The M lineage was suggested to have split from the O and C lineages in Asia Minor^3^, but our analyses recovered a poorly supported affinity with African subspecies. Thus, the origin of the M lineage is open to further investigation.
7. Future studies of honey bee origins should prioritise obtaining samples from northern Africa and the Middle East, as these regions are home to a high diversity of genetically diverse subspecies^46^. These samples will be especially important for testing the monophyly of the A lineage and the positions of the recently proposed S and Y lineages.

## Supplementary information

Supplementary file1

## Data Availability

The NCBI accession numbers of the sequences analysed are listed in the Supplementary Information. The analysed datasets and all output files are available online at Mendeley Data https://data.mendeley.com/datasets/fk9whcw3pp/1 (https://doi.org/10.17632/fk9whcw3pp.1).
