# Supplementary material for: Mitochondrial genomes illuminate the evolutionary history of the Western honey bee (Apis mellifera)
Source: Sci Rep. 2020 Sep 3;10:14515. doi: 10.1038/s41598-020-71393-0 (PMC7471700; doi:10.1038/s41598-020-71393-0)

Supplementary Information

**Mitochondrial genomes illuminate the evolutionary history of the Western honey bee (*Apis mellifera*)**

Erik Tihelka, Davide Pisani, Philip C. J. Donoghue & Chenyang Cai

**Contents**

**Table S1 |** Data set used for the phylogenetic, with NCBI accession for all sampled taxa ....................................................................................................................................................................................................2

**Table S2 |** Data partitioning scheme for the ML analyses …………................................................................................................3

**Figure S1 |** Analysis of the relationships among *Apis mellifera* subspecies with the site-homogeneous models based on the P12 dataset with branch lengths displayed. …………………………….…………….……………………………………………………………….…….………4

**Figure S2 |** Analysis of the relationships among *Apis mellifera* subspecies with the site-homogeneous models based on the P12 dataset with branch lengths omitted. ………………………………………………………………………………………………………………………….…5

**Figure S3 |** Analysis of the relationships among *Apis mellifera* subspecies with the site-homogeneous models based on the P12RNA dataset with branch lengths displayed. …………………………………………………………………………………………………..….……………6

**Figure S4 |** Analysis of the relationships among *Apis mellifera* subspecies with the site-homogeneous models based on the P12RNA dataset with branch lengths omitted. …………………..……………………………………………………………………………..…….……………7

**Figure S5 |** Analysis of the relationships among *Apis mellifera* subspecies with the site-homogeneous models based on the P123 dataset with branch lengths displayed. ………………..……………………………………………..….……………………………………………………8

**Figure S6 |** Analysis of the relationships among *Apis mellifera* subspecies with the site-homogeneous models based on the P123 dataset with branch lengths omitted. ……………………..………………………………..………..…………………………………………..……………9

**Figure S7 |** Analysis of the relationships among *Apis mellifera* subspecies with the site-heterogeneous Bayesian CAT-GTR+G model based on the P12 dataset with branch lengths displayed. …………………………….…………….……………………………………………………………………………………….………………………………………………..……10

**Figure S8 |** Analysis of the relationships among *Apis mellifera* subspecies with the site-heterogeneous Bayesian CAT-GTR+G model based on the P12 dataset with branch lengths omitted. …………………………….…………….………………………………………………………………………………………………………………………………………...……11

**Figure S9 |** Analysis of the relationships among *Apis mellifera* subspecies with the site-heterogeneous Bayesian CAT-GTR+G model based on the P12RNA dataset with branch lengths displayed. …………………………….…………….……………………………………………………………………………………………….…………………………………………..…12

**Figure S10 |** Analysis of the relationships among *Apis mellifera* subspecies with the site-heterogeneous Bayesian CAT-GTR+G model based on the P12RNA dataset with branch lengths omitted. …………………………….…………….……………………………………………………………………………………………….…………………………………………..…13

**Figure S11 |** Analysis of the relationships among *Apis mellifera* subspecies with the site-heterogeneous Bayesian CAT-GTR+G model based on the P123 dataset with branch lengths displayed. ………………………….…………….………………………………………………………………………………………………….………………………………………………14

**Figure S12 |** Analysis of the relationships among *Apis mellifera* subspecies with the site-heterogeneous Bayesian CAT-GTR+G model based on the P123 dataset with branch lengths omitted. ………………………….…………….………………………………………………………………………………………………….…………………………15

**Table S1**

Data set used for the phylogenetic, with NCBI accession for all sampled taxa.

| **Taxon** | **NCBI accession** |
| --- | --- |
| ***Western honey bee*** |  |
| *A. m. adansonii* | MN585109 |
| *A. m. capensis* | MG552691 |
| *A. m. carnica* | MN250878 |
| *A. m. carpatica* | AP018403 |
| *A. m. caucasica* | AP018404 |
| *A. m. iberiensis* | MN585110 |
| *A. m. intermissa* | KY926883 |
| *A. m. lamarckii* | KY464958 |
| *A. m. ligustica* | KX908209 |
| *A. m. meda* | KY464957 |
| *A. m. mellifera* | KY926884 |
| *A. m. monticola* | MF678581 |
| *A. m. sahariensis* | MF351881 |
| *A. m. scutellata* | MG552698 |
| *A. m. simensis* | MN585108 |
| *A. m. sinisxinyuan* | MN733955 |
| *A. m. syriaca* | KY926882 |
| *A. m. unicolor* | MN119925 |
|  |  |
| ***Outgroups*** |  |
| *A. cerana* | GQ162109 |
| *A. dorsata* | KX908207 |
| *A. florea* | JX982136 |

**Table S2**

Data partitioning scheme for the ML analyses.

| **Dataset** | **Partitioning scheme in nexus format** |
| --- | --- |
| ***P12*** | #nexus  begin sets;  charset Subset1 = 1-226 453-505 2053-2311 4623-4739;  charset Subset2 = 2312-2570 227-452 4944-5030 6488-7046 5480-5928 3644-3950;  charset Subset3 = 4857-4943 5929-6487 5031-5479 3337-3643 506-558;  charset Subset4 = 1601-1826 2571-2953 559-1079;  charset Subset5 = 1080-1600 2954-3336 1827-2052;  charset Subset6 = 3951-4286 7047-7223;  charset Subset7 = 4740-4856 7224-7400 4287-4622;  charpartition PartitionFinder = K81UF+I+G:Subset1, K81UF+I:Subset2, K81UF+G:Subset3, TIM+G:Subset4, F81+I:Subset5, HKY+I:Subset6, HKY+I:Subset7;  end; |
|  |  |
| ***P12RNA*** | #nexus  begin sets;  charset Subset1 = 1-226 453-505 2053-2311 4623-4739;  charset Subset2 = 1080-1600 2312-2570 227-452 2954-3336 1827-2052;  charset Subset3 = 506-558 4740-4856 7224-7400 4287-4622;  charset Subset4 = 1601-1826 2571-2953 559-1079;  charset Subset5 = 4857-4943 3337-3643 5031-5479 5929-6487 8840-9758;  charset Subset6 = 4944-5030 5480-5928 6488-7046 3644-3950;  charset Subset7 = 3951-4286 7047-7223;  charset Subset8 = 7401-8839;  charpartition PartitionFinder = K81UF+I+G:Subset1, K81UF+I:Subset2, HKY+I:Subset3, TIM+G:Subset4, K81UF+G:Subset5, TIM+I:Subset6, HKY+I:Subset7, GTR+I+G:Subset8;  end; |
|  |  |
| ***P123*** | #nexus  begin sets;  charset Subset1 = 2401-3078 3856-5004 838-2400 3079-3855 1-678;  charset Subset2 = 10570-11100 679-837 6934-7284 5926-6933;  charset Subset3 = 8893-10569 5005-5925 7285-7545 7546-8892;  charpartition PartitionFinder = GTR+I:Subset1, HKY+I+G:Subset2, TIM+I+G:Subset3;  end; |

**Figure S1**

Analysis of the relationships among *Apis mellifera* subspecies with the site-homogeneous models based on the P12 dataset with branch lengths displayed


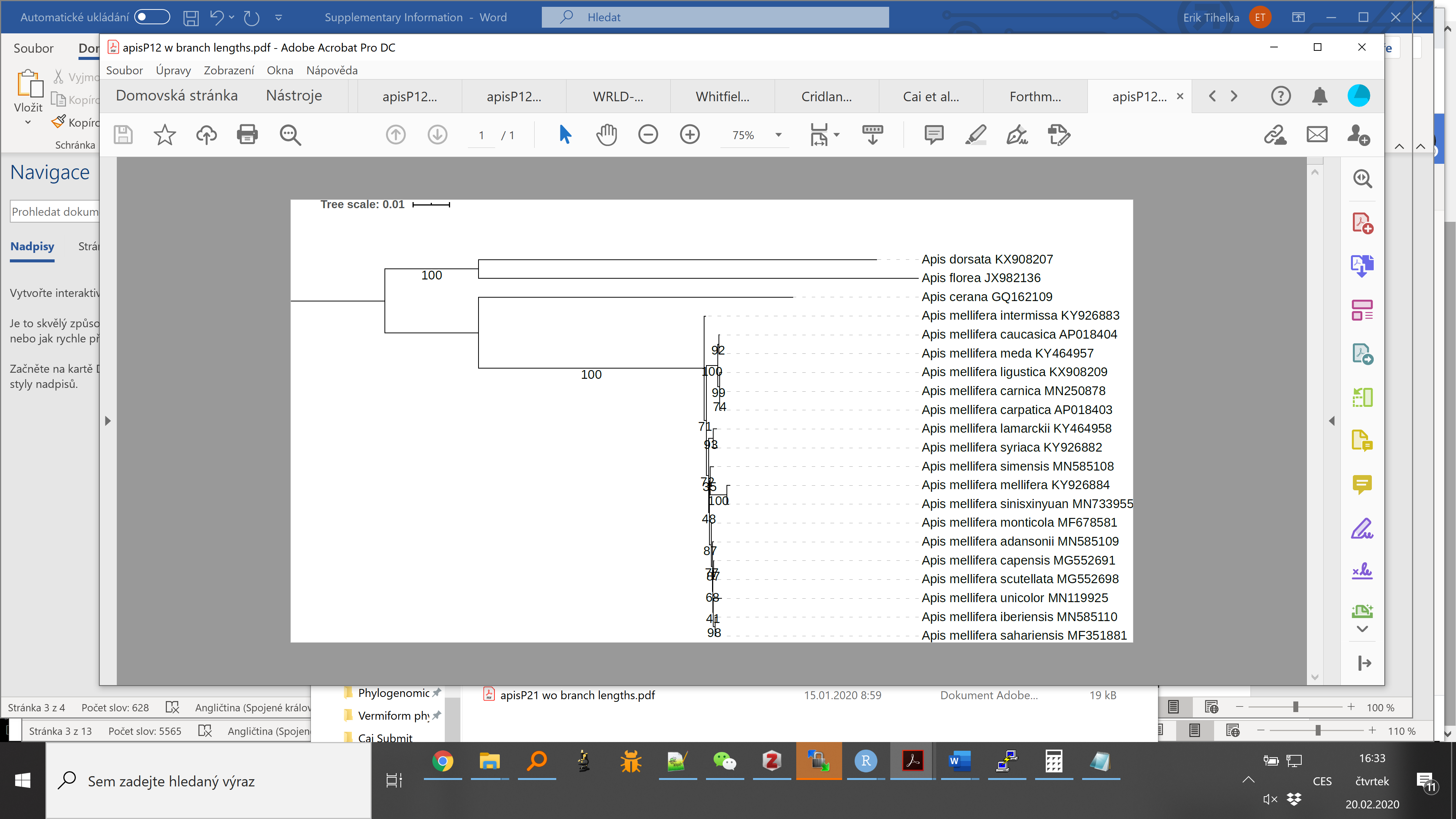


**Figure S2**

Analysis of the relationships among *Apis mellifera* subspecies with the site-homogeneous models based on the P12 dataset with branch lengths omitted


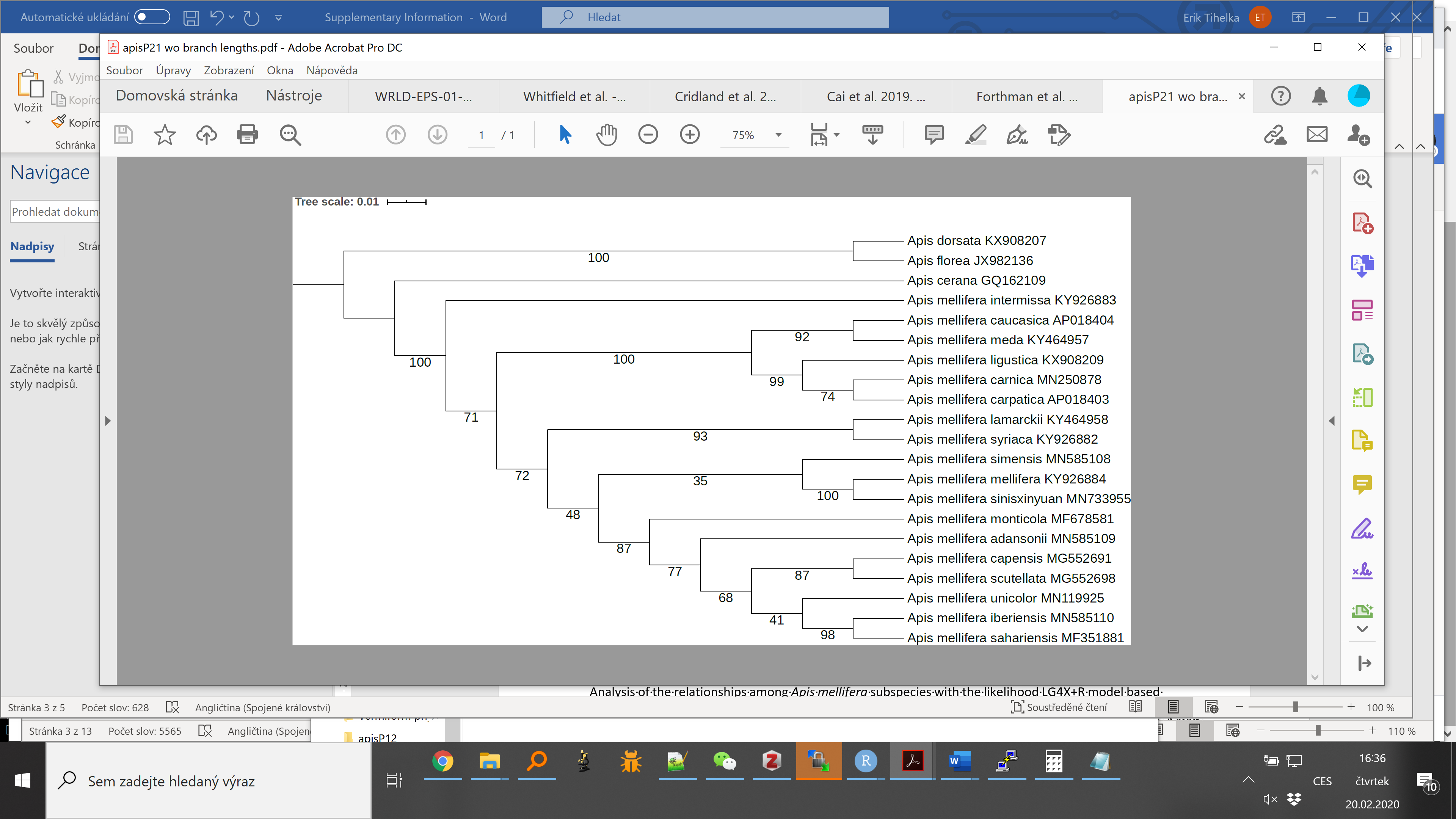


**Figure S3**

Analysis of the relationships among *Apis mellifera* subspecies with the site-homogeneous models based on the P12RNA dataset with branch lengths displayed


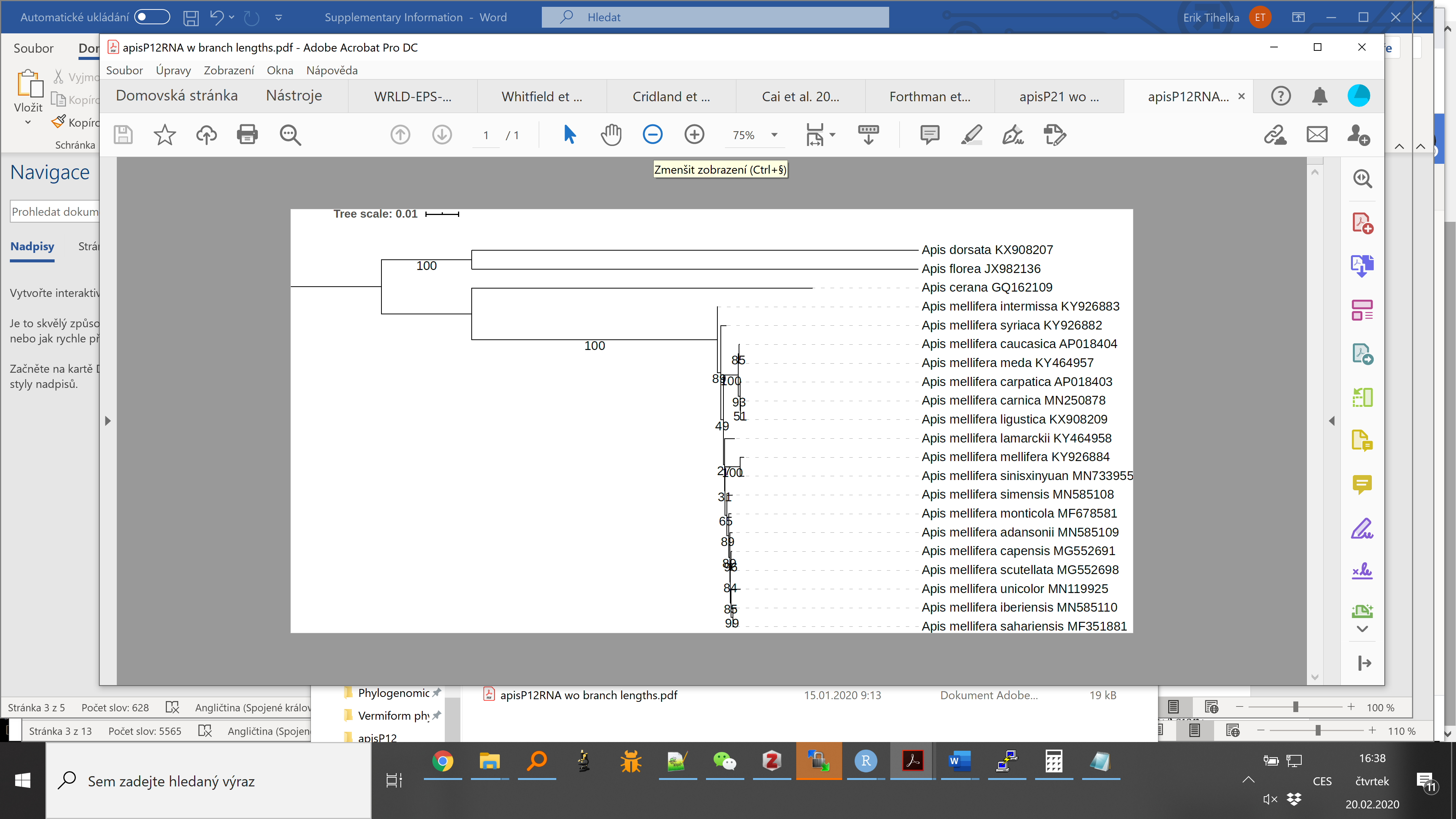


**Figure S4**

Analysis of the relationships among *Apis mellifera* subspecies with the site-homogeneous models based on the P12RNA dataset with branch lengths omitted


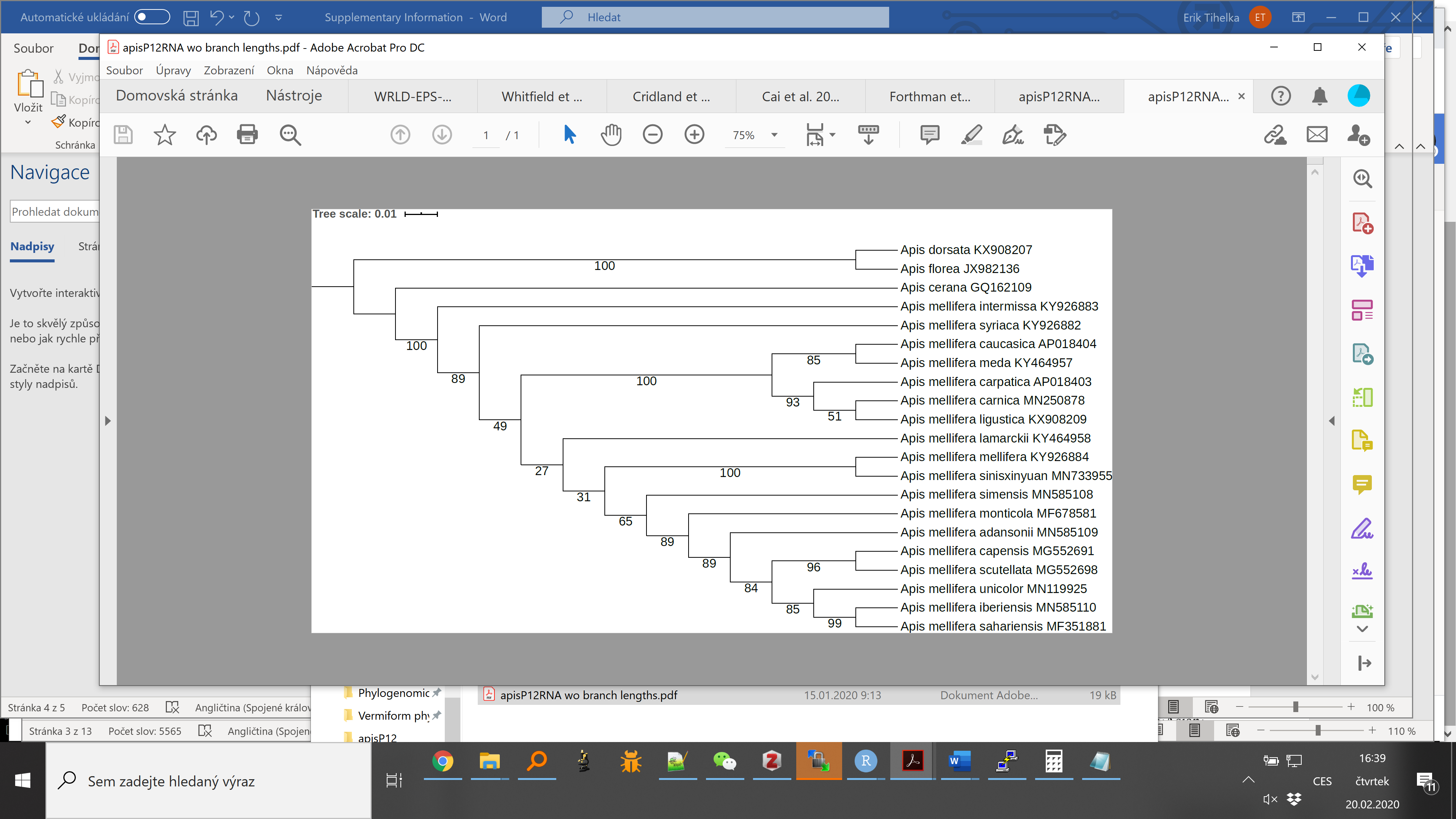


**Figure S5**

Analysis of the relationships among *Apis mellifera* subspecies with the site-homogeneous models based on the P123 dataset with branch lengths displayed


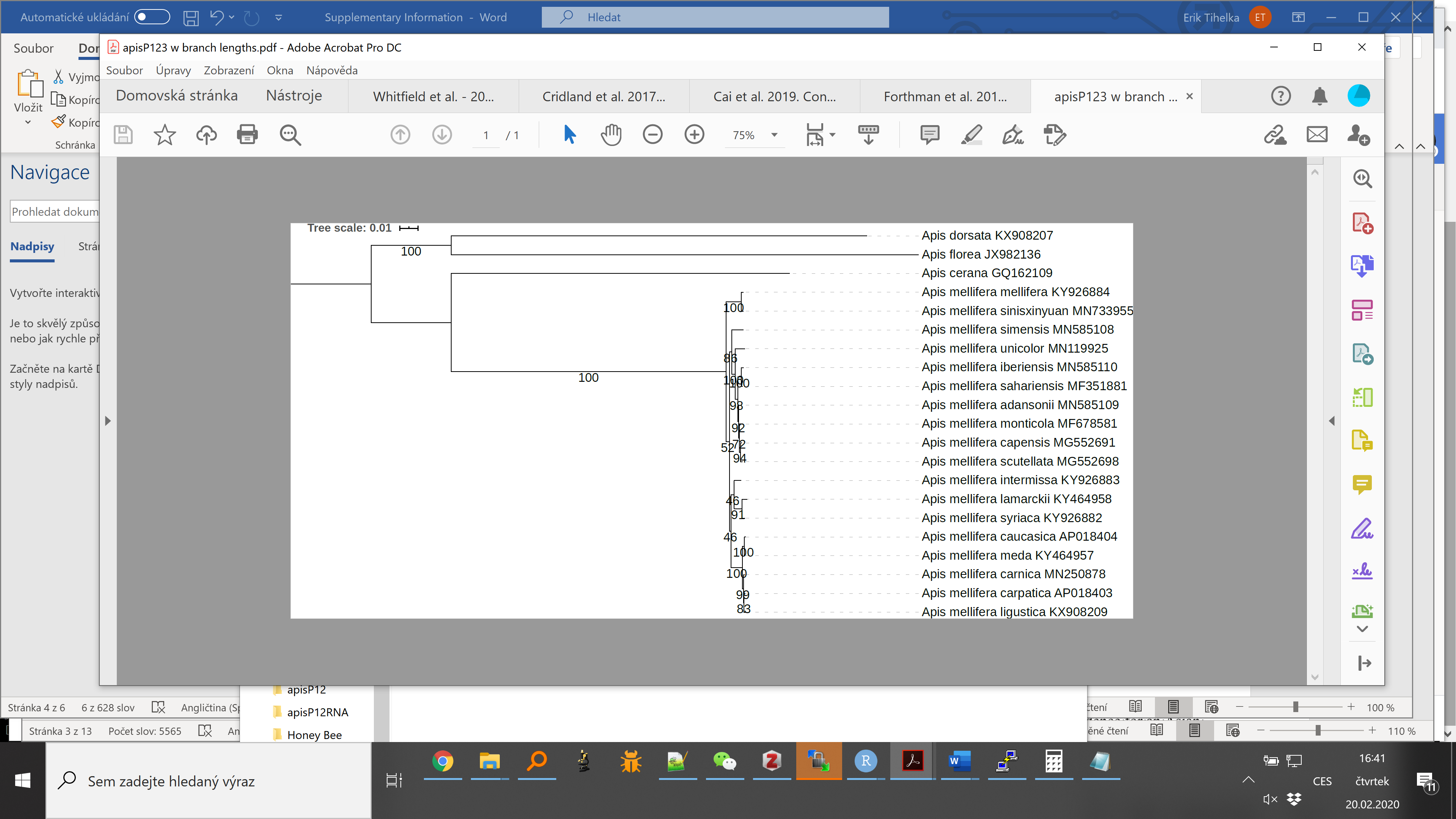


**Figure S6**

Analysis of the relationships among *Apis mellifera* subspecies with the site-homogeneous models based on the P123 dataset with branch lengths omitted


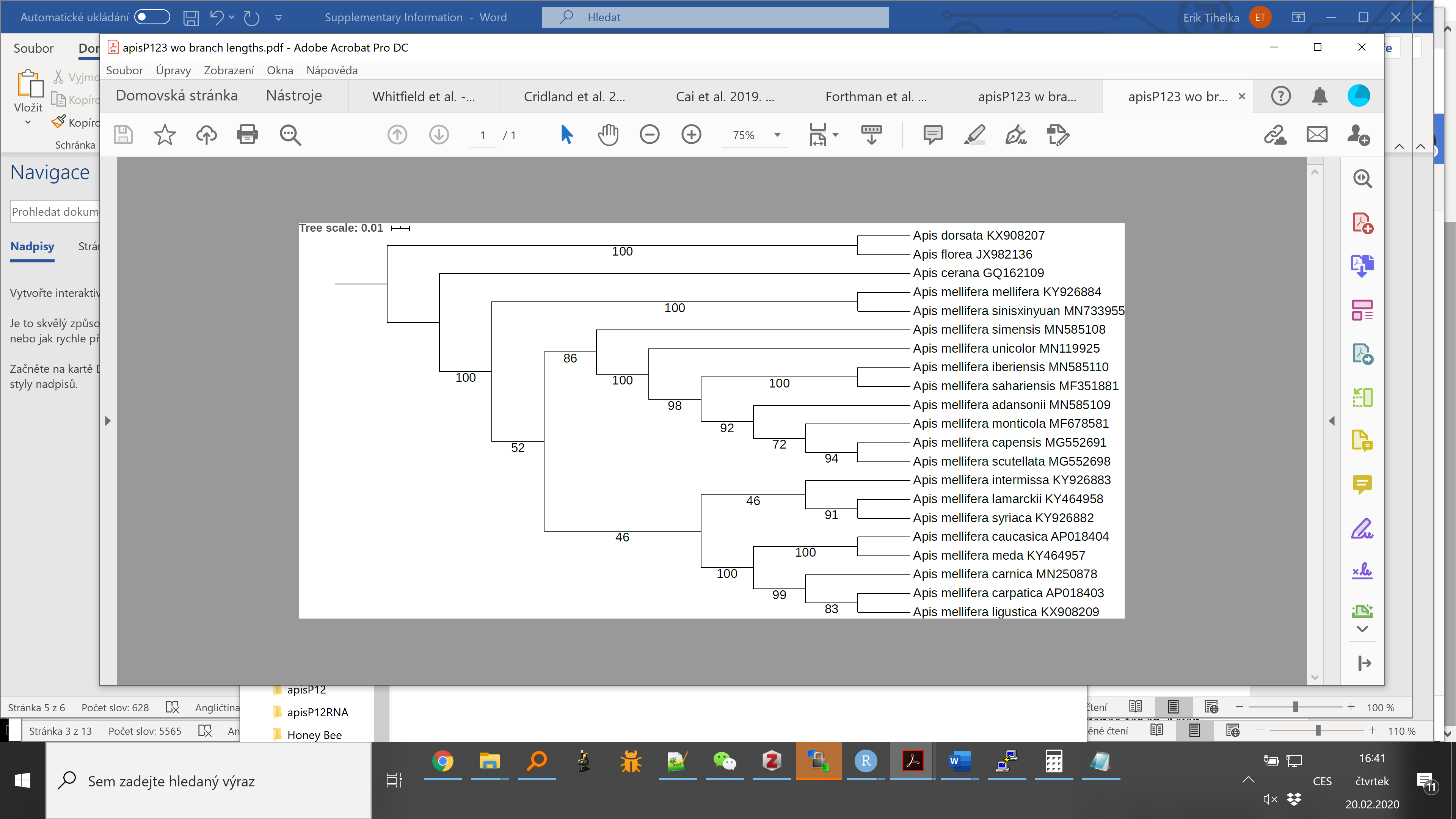


**Figure S7**

Analysis of the relationships among *Apis mellifera* subspecies with the site-heterogeneous Bayesian CAT-GTR+G model based on the P12 dataset with branch lengths displayed.


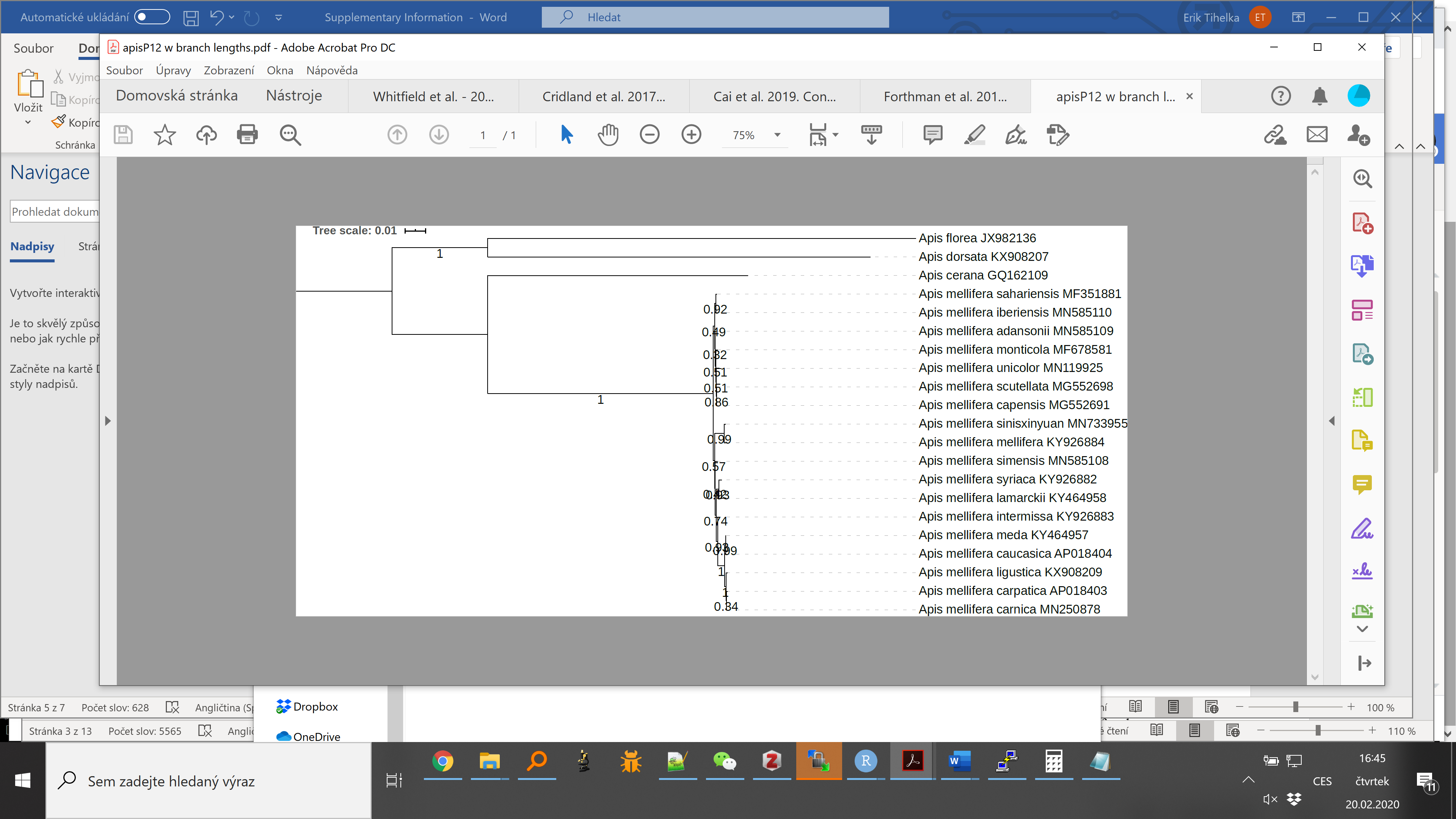


**Figure S8**

Analysis of the relationships among *Apis mellifera* subspecies with the site-heterogeneous Bayesian CAT-GTR+G model based on the P12 dataset with branch lengths omitted.


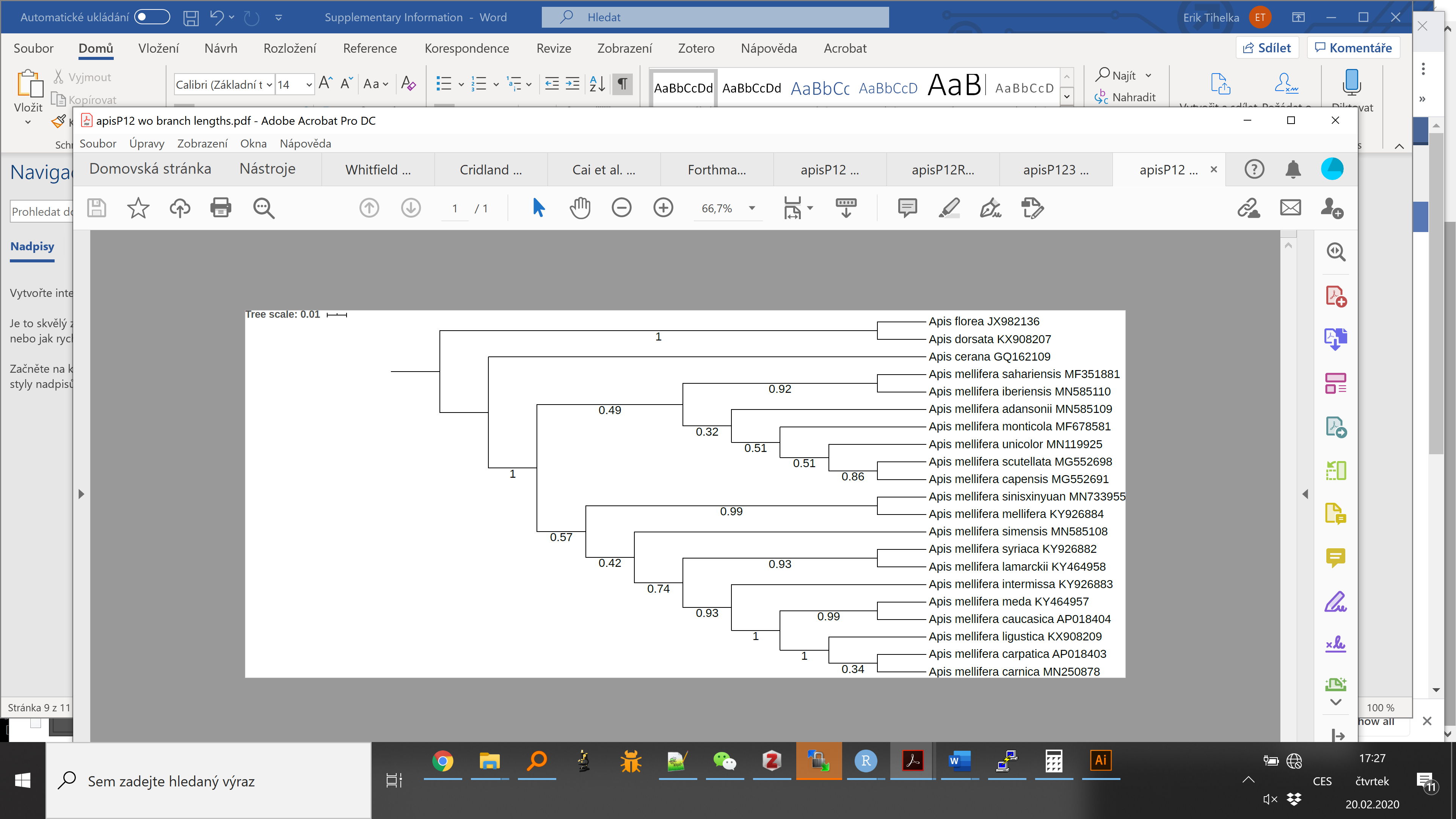


**Figure S9**

Analysis of the relationships among *Apis mellifera* subspecies with the site-heterogeneous Bayesian CAT-GTR+G model based on the P12RNA dataset with branch lengths displayed.


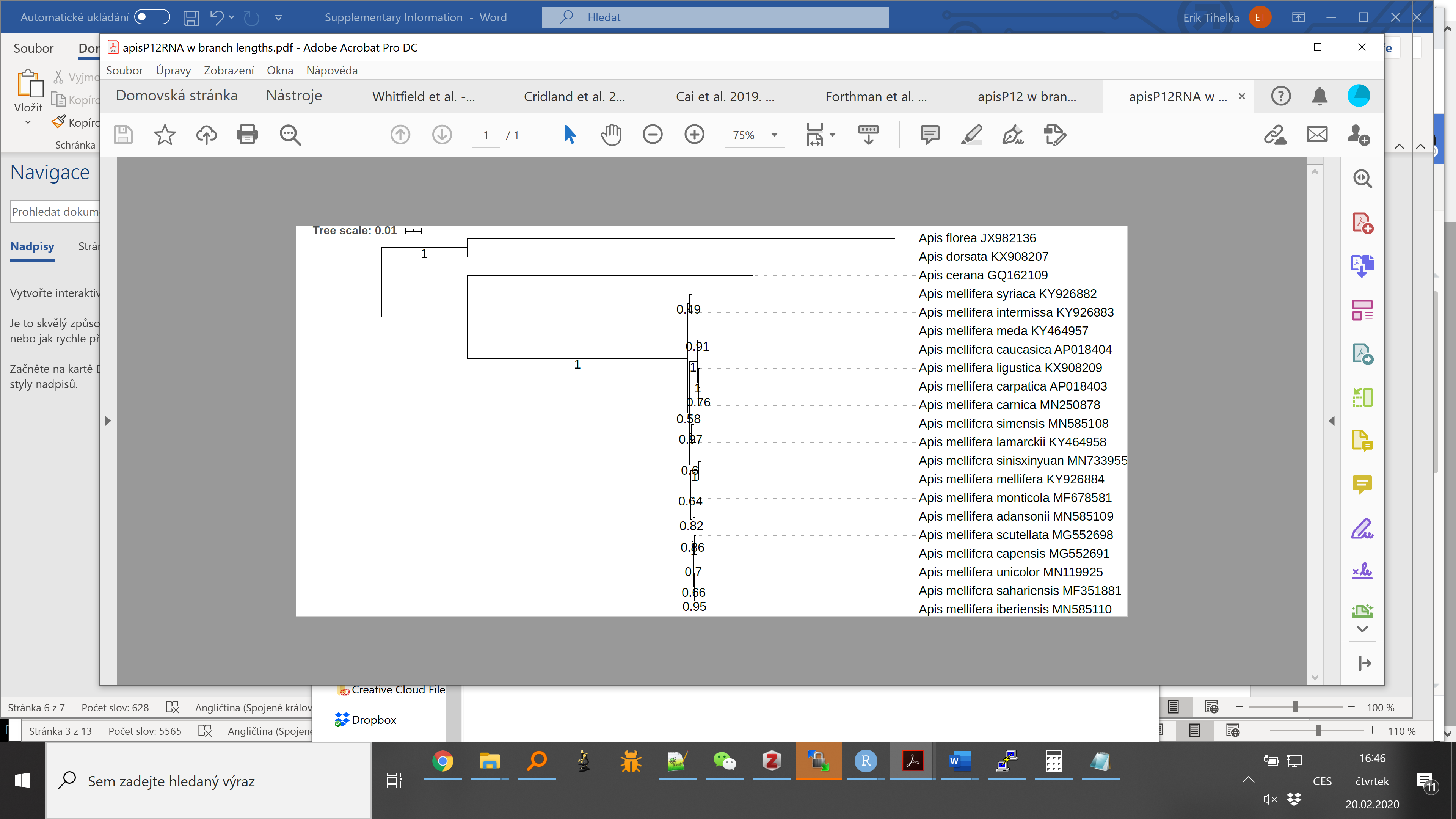


**Figure S10**

Analysis of the relationships among *Apis mellifera* subspecies with the site-heterogeneous Bayesian CAT-GTR+G model based on the P12RNA dataset with branch lengths displayed.


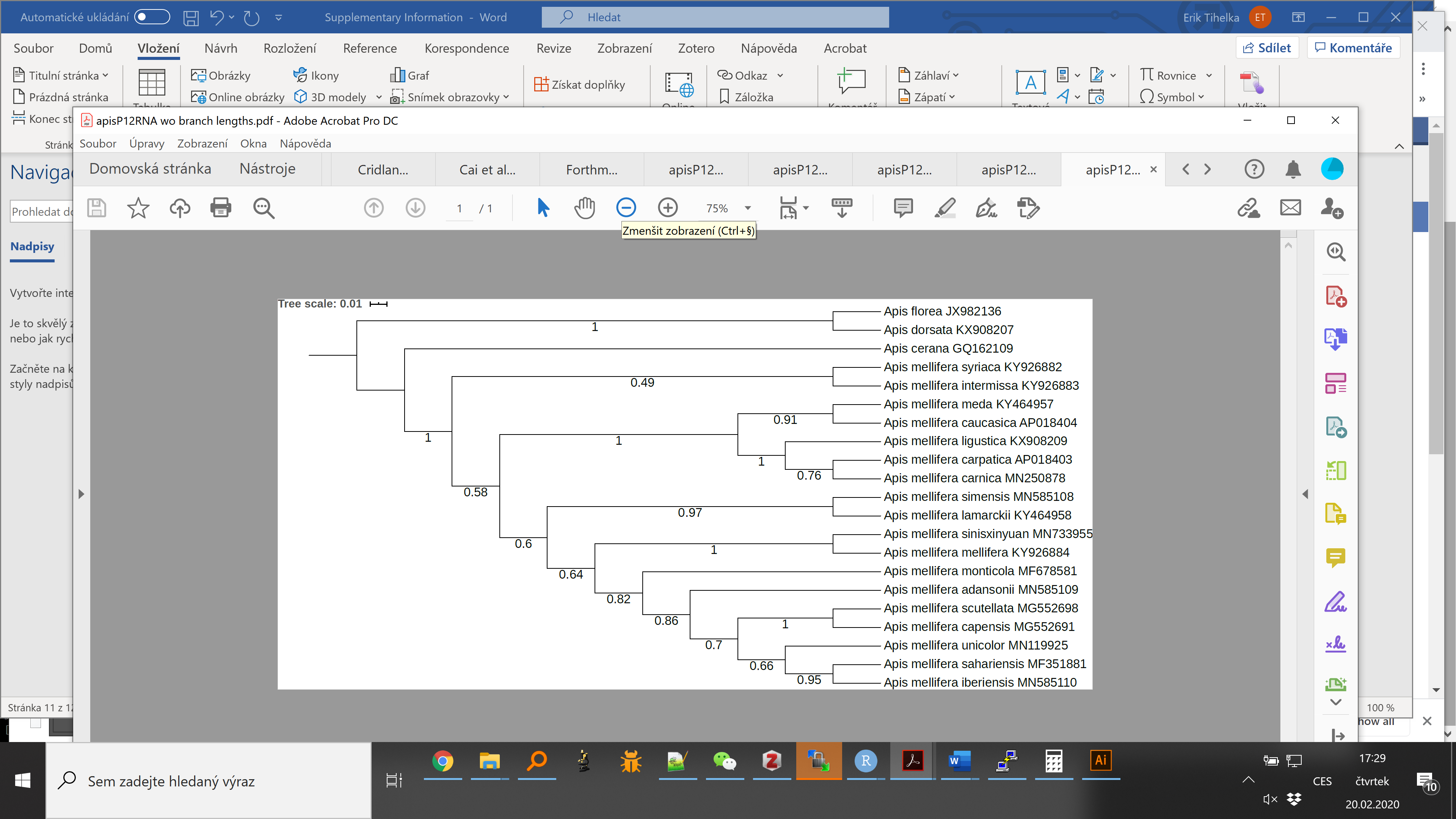


**Figure S11**

Analysis of the relationships among *Apis mellifera* subspecies with the site-heterogeneous Bayesian CAT-GTR+G model based on the P123 dataset with branch lengths displayed.


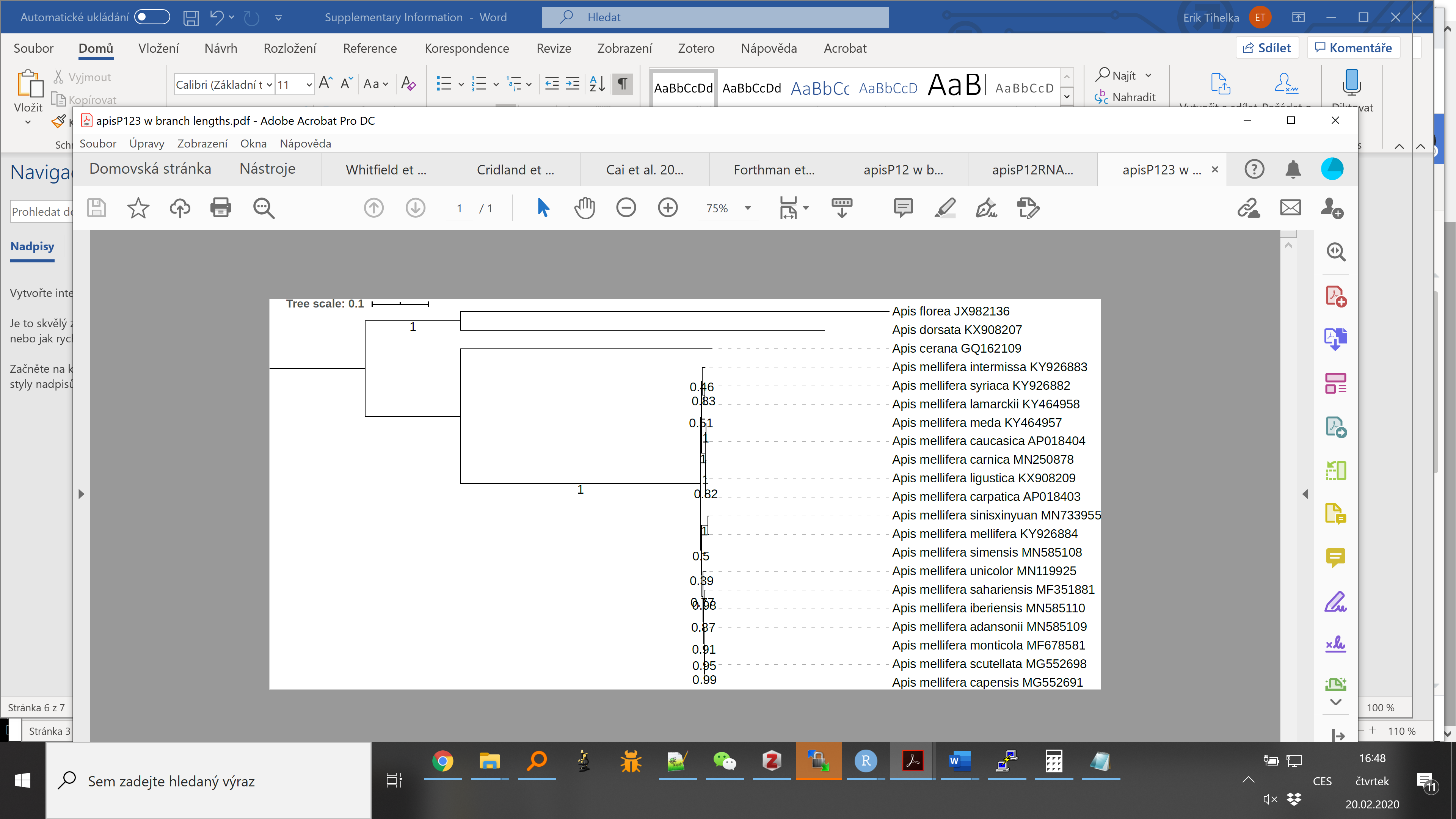


**Figure S12**

Analysis of the relationships among *Apis mellifera* subspecies with the site-heterogeneous Bayesian CAT-GTR+G model based on the P123 dataset with branch lengths omitted.


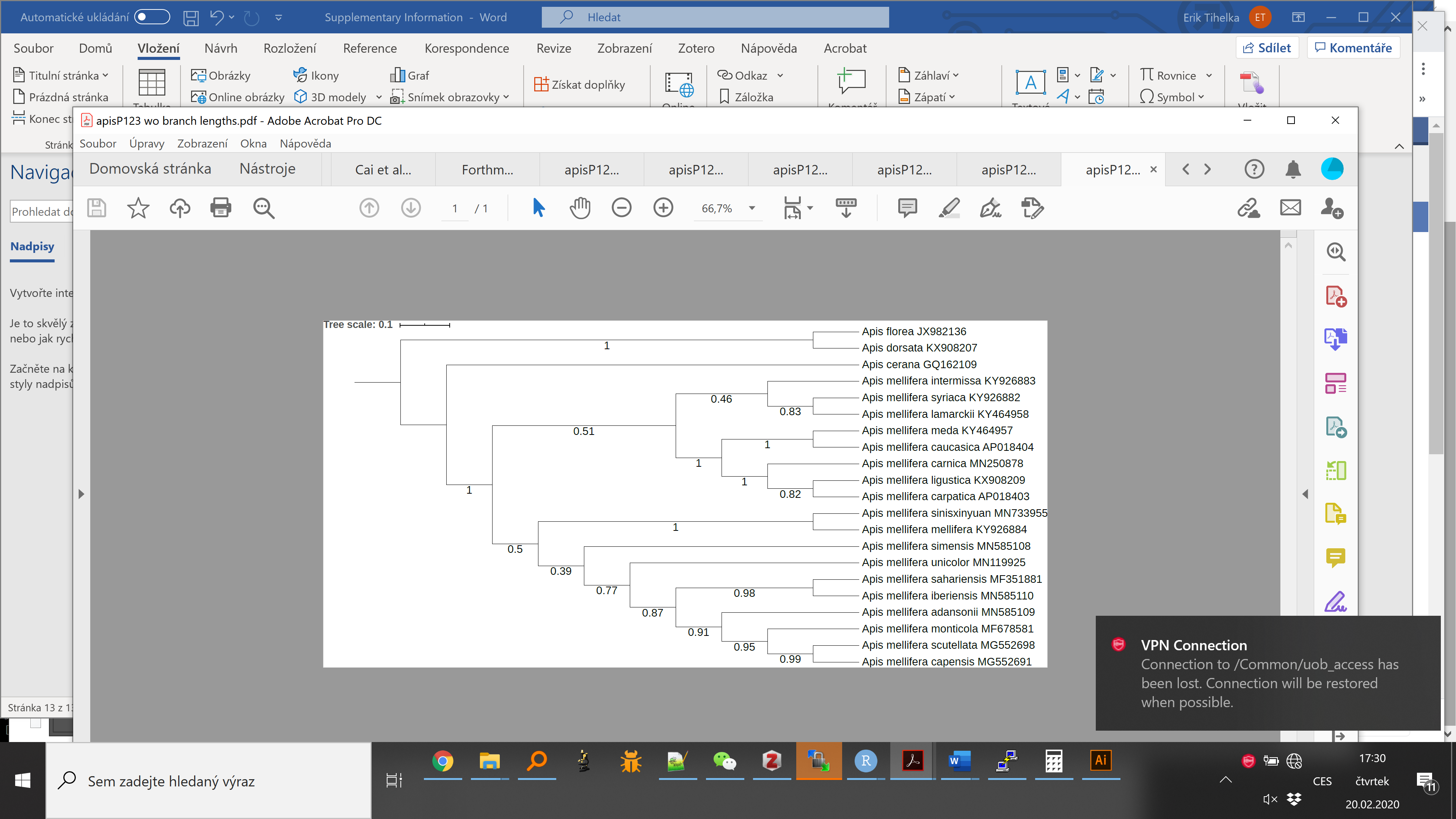

Supplement: Supplementary file 1 — Supplementary file1 [file 41598_2020_71393_MOESM1_ESM.docx]
